# What drives soil degradation after gravel mulching for 6 years in northwest China?

**DOI:** 10.3389/fmicb.2023.1224195

**Published:** 2023-07-21

**Authors:** Yang Qiu, Xingyi Chen, Yajun Wang, Yubao Zhang, Zhongkui Xie

**Affiliations:** Northwest Institute of Eco-Environment and Resources, Chinese Academy of Sciences, Lanzhou, China

**Keywords:** gravel mulch, soil microbial community, soil carbon, long-term, soil degradation

## Abstract

Gravel mulch is an agricultural water conservation practice that has been widely used in the semi-arid region of northwest China, but its effectiveness is now lessening due to soil degradation caused by long-term gravel mulching. In this study, we report on a 6-year-long gravel mulch experiment conducted in the northwestern Loess Plateau to evaluate the impact of gravel mulch on soil physicochemical properties and microbial communities, with the objective of clarifying the causes of long-term gravel mulching-induced land degradation. After 6 years mulching, we found that gravel mulched soil contained significantly higher concentrations of total carbon and total organic carbon than non-mulched soil (control). Long-term gravel mulching significantly changed the soil microbial diversity and abundance distribution of bacterial and fungal communities. Notably, the relative abundance of *Acidobacteria* was significantly higher under gravel mulching than the control (no mulching), being significantly greater in the AG treatment (small-sized gravel, 2–5 mm) than all other treatments. Conversely, the relative abundance of *Actinobacteria* was significantly lower under gravel mulching than the control, being the lowest in the BG treatment (large-sized gravel, 40–60 mm). At the same time, the relative abundance of *Bacteroidetes* was significantly lower in AG yet higher in BG vis-à-vis the other treatments. Of the various factors examined, on a 6-year scale, the capture of dust by gravel mulch and altered carbon and nitrogen components in soil play major contributing roles in the compositional change of soil microorganisms. These results suggest that modified soil material input from gravel mulching may be the key factor leading to soil degradation. More long-term experimental studies at different sites are now needed to elucidate the mechanisms responsible for soil degradation under gravel mulching.

## 1. Introduction

Gravel mulching refers to practices aiming at reducing soil evaporation, maintaining soil temperature, and increasing rainfall utilization in arid and semi-arid areas, by applying a layer of gravel of particular thickness and particle diameter upon the soil surface. The technique widely used in farmlands, greening projects, padding rail ties, and permafrost engineering because of its simplicity, low cost, and long-lasting effects. In arid and semi-arid areas of northwest China, gravel mulching has been used for more than 300 years in agricultural land (Wang et al., 2004). In some basins in the northwest Loess Plateau, farmland with gravel mulching accounts for more than 80% of the total farmland area (Chen et al., 2008). In China’s Gansu province, gravel mulched farmland totaled 72,327 ha at the end of 2016, about 2.4 times its area in 1949 according to the “Gansu Rural Yearbook” (2017). As of 2019, the amount of gravel mulched farmland in Ningxia province in central China exceeded 6,700 ha, a 10-fold increase compared to 2003 (Yu et al., 2019). In addition, gravel mulching techniques are being increasingly applied to urban greening and wilderness vegetation restoration in arid and semi-arid regions, in which gravel layer coverings are placed around greening trees and wilderness shrubs to mitigate soil erosion by wind and water (Qiu et al., 2021). Furthermore, due to the differences in the mechanics and hydrothermal processes of gravel and soil, gravel mulching is widely used in padding rail ties or permafrost engineering (Wen et al., 2007; Zhang et al., 2017).

Previous research has shown that the hydrothermal effects of gravel mulching can vary significantly at different gravel size and timescales. As the mulching life lengthens, the moisture retention performance decreases, the soil begins to tighten up, soil nutrient conversion slows down, and fertility declines (Qiu et al., 2015). Although this degradation typically occurs after 15 to 20 years of prolonged mulching, it still poses problems for the spread of gravel mulched farmland. This long-term mulching has a significant impact on soil microbes and may play a larger role in the degradation of gravel-covered farmland (Qiu et al., 2017). Surprisingly, however, the main driving factors of soil microbial changes under gravel mulching have yet to be investigated. Previous studies did report that the size of the gravel particles is of importance in reducing the soil evaporation and surface runoff, which consequently should also change soil microecology (Qiu et al., 2014, 2021). The combination of grit and soil intersections could impair processes of water and heat regulation after prolonged mulching (Wang et al., 2010), and that dust deposition may substantially affect the properties of soil under a cover of gravel (Li et al., 2001). However, the mechanism by which gravel mulch with different-sized particles influences soil microorganisms on relatively long time scales has yet to be studied in depth. This presents a key issue pertinent to sustainable land use and soil conservation in arid and semi-arid areas whose scientific study is imperative for providing a theoretical basis for improved gravel mulching management and land degradation protection.

Here, a 6-year-long gravel mulch experiment on the Loess Plateau of northwest China was conducted to investigate the effects of applying gravel mulch of two different sizes on soil physicochemical properties and soil microbial community structure and diversity. Hence, the objectives of this study were (1) to determine the differential impact of 6 years of gravel mulching with the different-sized particles on soil physicochemical properties and soil microbial diversity; (2) to evaluate correlations between soil physicochemical properties and soil microbial changes under gravel mulching; and (3) to explore the key factors affecting soil degradation in response to 6 years of gravel mulching.

## 2. Materials and methods

### 2.1. Field site and experimental design

The experiment was conducted at the Gaolan Ecology and Agriculture Research Station, of the Northwest Institute of Eco-Environment and Resources, Chinese Academy of Sciences. This station lies in the northwestern part of the Loess Plateau (Gaolan County, Lanzhou City, Gansu Province: 36°13′′N, 103°47′′E), at an elevation of 1800 m a.s.l. Mean annual rainfall is 327 mm, of which nearly 70% comes between June and September, and the mean annual air temperature is 8.4°C, with monthly minimum and maximum means of −9.1°C (January) and 20.7°C (July). Here, the zonal soil types are derived from ash–calcium and loess parent material, and the predominant zonal vegetation is desert grassland.

### 2.2. Experimental design

To study the key factors affecting soil microbial changes under gravel mulching in the Loess Plateau, three different treatments were established in 2015, in triplicate, consisting of soil mulched with small-sized gravel (2–5 mm dimension, AG), large-sized gravel (40–60 mm dimension, BG), and the control treatment with no mulching (CK). All nine replicate plots (each 3 m × 5 m) were randomly distributed. Gravel samples of different sizes, consisting mainly of silica, were obtained from fluvial material from the Yellow River. All plots had an approximately 100% surface cover of gravel (AG or BG) that was 10-cm thick; this thickness and the corresponding particle size range are commonly applied by farmers in the northwestern Loess Plateau. No plants were planted during the experiment.

### 2.3. Soil sampling

Before taking soil samples, the gravel cover layer on the soil surface should be removed first. In each plot, 10 soil samples were randomly collected from a 0–20 cm depth using a soil auger on 25 September 2021. These soil samples were then pooled per plot into one homogenized composite sample. Plant tissues and stones were removed using a 2-mm sieve, after which each sieved sample was divided into two parts. One part was stored in a container with 15 kg of dry ice and sent to the Biomarker Technologies Corporation, Beijing, China (www.biomarker.com.cn) for assessing its microbial community structure via amplicon sequencing, while the other part was air-dried for its soil physicochemical analysis.

### 2.4. Determination of soil physicochemical properties and extraction of DNA

Soil organic carbon was determined by potassium dichromate method (Bao, 2008), while total carbon (TC) and total nitrogen (TN) was measured by an elemental analyzer (Vario Macro, Elementar, Germany). Available nitrogen (AN) was determined by the NaOH alkali diffusion method, with the nitrate-N and ammonia-N contents of soil samples measured by ion chromatography (ICS-600, Thermo Fisher Scientific, the United States of America). Soil pH was quantified according to ISO 10390 (ISO, 1994): 5 grams of soil were shaken for 5 min at 25 mL 1 M KCl and allowed to stand for 2 h; pH was then recorded using glass electrodes. Total salt content of soil was determined by the conduction method (Lu, 1999). Dust accumulation (DA) in the gravel mulch layer was assessed using an iron sampling grid (1 m × 1 m) positioned at five points within each plot; dust on the stone surface was washed away by pure water and then oven-dried the solution for 12 h. Particle distribution of dust accumulation was measured by a laser particle size analyzer (Ambivalue BV, Dussen, The Netherlands). Total amount of genomic DNA was extracted from each soil sample by using the PowerSoil DNA Isolation Kit (MoBio Laboratories, Inc., USA), as directed by the manufacturer, and stored at −80°C for subsequent analyses.

### 2.5. PCR amplification, sequencing, and statistical analysis

The MiSeq sequencing strategies used in this study for bacteria and fungi followed the respective protocols described by Kozich et al. (2013). The 16S rRNA tag-encoded high-throughput sequencing was carried out on the Illumina MiSeq platform by Biomarker Technologies Co., Ltd., Beijing, China. Pairs of reads from the original DNA fragments were aligned according to previously described methodology (Magoč and Salzberg, 2011). Sequencing reads were assigned to each sample according to their unique barcode, and all sequences were analyzed with QIIME (Quantitative Insights Into Microbial Ecology) software and the UPARSE pipeline. The reads were first underwent quality-filtering by applying the method available in QIIME, whose default settings were then used for their Illumina processing. The UPARSE pipeline was then used to identify operational taxonomic units (OTUs) at 97% similarity threshold. For each OTU, a representative sequence was selected for its taxonomic assignment using the RDP Classifier. Next, the estimated species richness was identified by a rarefaction analysis; the Chao1, ACE，Simpson, and Shannon indices of diversity were obtained as described previously (Schloss et al., 2009).

Statistical analyses and testing were implemented in SPSS v19.0 software (IBM, Armonk, NY, United States) and using the Rstudio v4.2.0 software (R Foundation for statistical Computing, Vienna, Austria). Data were subjected to one-way analysis of variance (ANOVA); when an ANOVA’s F-ratio test was significant, the means of each treatment were compared using Fisher’s least significant difference test, with statistical significance set at *p* < 0.05.

## 3. Results and Discussion

### 3.1. Effects of 6-year of gravel mulching on soil physicochemical properties

**Figure 1:**
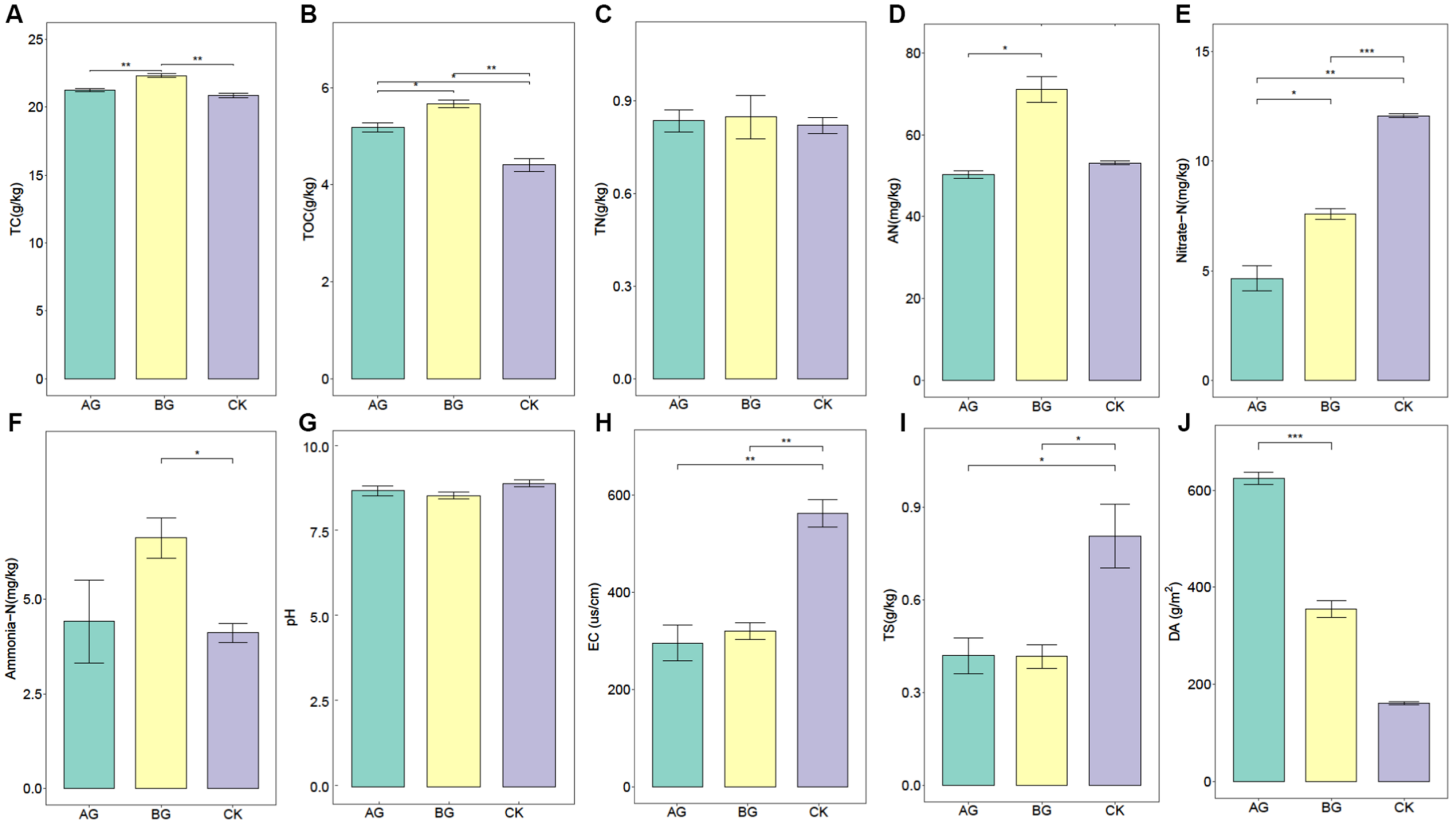
Soil physicochemical properties measured in the 0–20 cm soil layer and dust accumulation in gravel mulch layer of the three treatments. CK denotes the treatment without gravel mulch (control), while AG and BG denote treatments with 2–5 mm and 40–60 mm particles used as gravel mulch, respectively. TC, total carbon **(a)**; TOC, total organic carbon **(b)**; TN, total nitrogen **(c)**; AN, available nitrogen **(d)**; Nitrate-N, Nitrate Nitrogen **(e)**; Ammonia-N, Ammonia nitrogen **(f)**; pH **(g)**; EC, electrical conductivity **(h)**; TS, total salt **(i)**; DA, dust accumulation **(j)**. The *, **, and *** respectively indicate a significant difference between means at *p* < 0.05, *p* < 0.01, and *p* < 0.001.

Figure 1 shows the TC and TOC in the variously treated soils. Compared with the control, TC in the AG or BG treatment averaged 21.24 and 22.33 g/kg, respectively. TC in the BG treatment increased 7.1% after 6 years of gravel mulching compared with the control, being the highest point among all treatments (Figure 1A). A similar trend was found for the TOC content (Figure 1B). Although there was no significant difference in the total nitrogen (TN) content among treatments, the available nitrogen (AN) content was higher under the BG than AG treatment. At the same time, ammonia-N content of the BG treatment was significantly higher than that of the control. By contrast, for the nitrate-N content, it was significantly lower in both mulching treatments than the control, especially in the AG treatment.

There was no significant difference in soil pH after 6 years of gravel mulching, but the total salt (TS) content was 47.9 and 48.3% lower for both AG and BG treatments, along with significantly lower EC values, in comparison with the control. We also found a significant change in the accumulated dust in the gravel mulch layer after 6 years of mulching, with the DA of the AG treatment being significantly higher than that of the other two treatments.

Changes to the TC, TOC, and AN in soil and significant increases in crop yields on gravel-covered farmlands during the same cover time from other researchers’ papers are mutually verifiable. For example, watermelon yields were significantly higher after 5 years of gravel mulching than at the onset mulching (Liang et al., 2011). Since the gravel mulch layer covering the soil leads to the isolation of material inputs such as litter, significant changes in soil physico-chemical properties may be due to several factors: (i) Gravel mulching can significantly increase soil moisture and reduce soil erosion, which is well recognized as a serious problem in the Loess Plateau (Li et al., 2000; Wang et al., 2009; Lv et al., 2019). Our previous research showed that the amendments with gravel significantly increased soil moisture, the soil moisture of AG, BG, and CK treatment were 19.1, 19.8, and 13.6%, respectively, after 6-year gravel mulching. Applying gravel mulching also significantly increased the rainfall threshold to generate runoff, and caused fewer runoff events (Qiu et al., 2021). (ii) Gravel mulch collects atmospheric dust; previous research has found that gravel mulch is effective at capturing wind dust, and there is 2–3 times more organic matter in the air than local soils of the Loess Plateau (Li et al., 2001). The changed nitrate-N and ammonia-N contents suggest that gravel mulching may inhibit the nitrification process in the soil, leading to less nitrate-N, or that more it is simply washed away by soil runoff under gravel mulching (Qiu et al., 2021).

Gravel mulching reduced the TS and EC values of soil, a result in line with other findings that suggested gravel mulching could alter the water–salt transport process, prompting some to apply it to saline land management (Sun et al., 2013). This is due to the fact that salt transport in soil is dominated by water, yet gravel mulching increases the rate of infiltration (Lv et al., 2021); hence, as soil salt moves downwards with water infiltration and gravel mulching reduces soil water evaporation (Qiu et al., 2014), less salt accumulates in the upper layer as water evaporates. Concerning the impact of gravel mulch on DA, this is best explained by the fact that the mulch is essentially a porous medium with abundant porosity; accordingly, the AG treatment has a larger specific surface area and can therefore accommodate a greater DA. Albeit at static measure—only indicating the DA in the gravel layer at some time point (i.e., after 6 years of mulching)—it suggests that most of the atmospheric dust captured by the gravel layer seeps into the soil through precipitation solubility, which may have contributed to the changed carbon and nitrogen contents of the experimental soil. Furthermore, previous research has shown that the size of the gravel particles is of importance in changing the channel characteristics of water vapor transport in the gravel mulch layer, which consequently should also change soil water erosion and the loss of important nutrients such as carbon and nitrogen (Li et al., 2000; Qiu et al., 2014, 2021).

From the results of this study, we believe that the significant improvement in the ability of gravel mulching to capture atmospheric dust is one of the main reasons for the increase in soil carbon and nitrogen content after 6-years mulching. In addition, our previous research found gravel mulch significantly reduces runoff caused by rainfall, which may also be one of the reasons for the increase in soil carbon and nitrogen after 6-years mulching (Qiu et al., 2021). As for the decrease in soil carbon and nitrogen content after longer years, we believe that there may be two main reasons. One is that gravel mulching significantly increases the infiltration rate of rainfall, which may take away nutrients from the surface soil (Lv et al., 2021). Secondly, soil microorganisms may play an important role. But the specific mechanism still needs more long-term studies.

### 3.2. Soil mechanical composition and residual atmospheric dust

**Figure 2:**
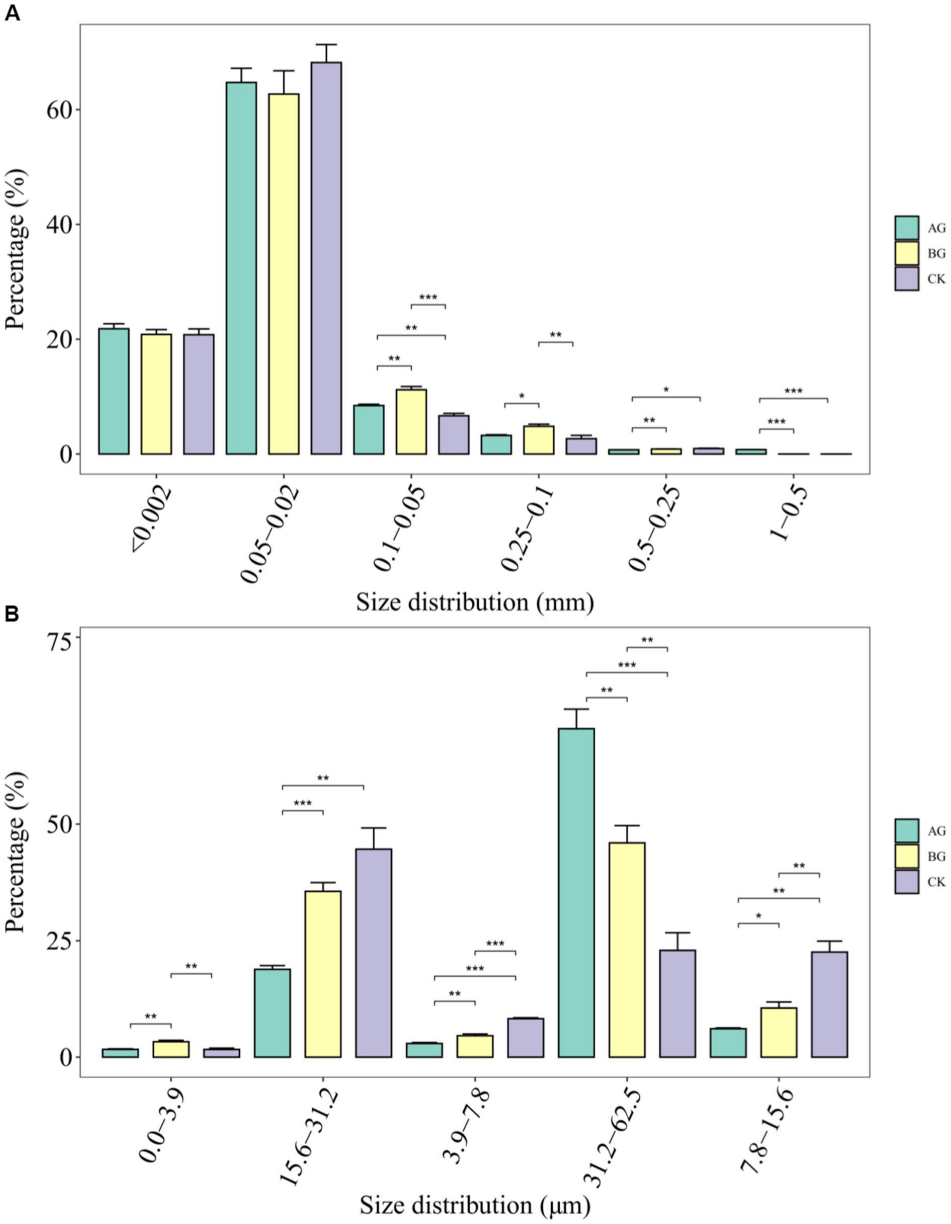
Soil mechanical composition **(A)** and particle distribution of dust accumulation **(B)** in the gravel mulch layer of different treatments. The *, **, and ***, respectively, indicate a significant difference between means at *p* < 0.05, *p* < 0.01, and *p* < 0.001. CK denotes the treatment without gravel mulch (control), while AG and BG denote treatments with 2–5 mm and 40–60 mm particles used as gravel mulch, respectively.

Evidently, the 6 years of gravel mulching had an impact on soil mechanical composition (Figure 2). The percentage of 0.1–0.05 mm soil particles was significantly greater under the BG treatment than the two other treatments, with that under the AG treatment significantly surpassing the control as well. Another study had suggested that the combined gravel and soil interface, which causes some minute gravel particles to enter the soil surface and affect both the warming of soil and its water retention function, is one of the main reasons why soil degradation ensues under gravel mulch (Wang et al., 2010). This view is consistent with our results, especially for the small-particle gravel mulching treatment (AG), where the gravel mulching layer gradually compresses subsoil gravel into the soil, while precipitation processes increasingly obscure the gravel mulching layer and soil boundary. In this study, we also measured the particle distribution of residual atmospheric dust in the gravel mulch layer, finding significant differences among the treatments, of which the BG treatment containing a significantly greater proportion of 0–3.9 μm particles. For the 3.9–31.2 μm size range, its percentage of DA under both mulching treatments was significantly lower than that of the control. However, for the 31.2–62.5 μm range, its percentage of DA was significantly higher in the AG treatment than the two other treatments, and also significantly higher in the BG treatment than the control. DA on the gravel surface was significantly correlated with larger-sized portions of soil mechanical composition. This suggest the latter’s variation may be due to a combination of subgrade gravel mixing and the selective dust capture ability of layers of different sized gravel mulch.

### 3.3. Effects of 6-year gravel mulching on soil microbial diversity

**Figure 3:**
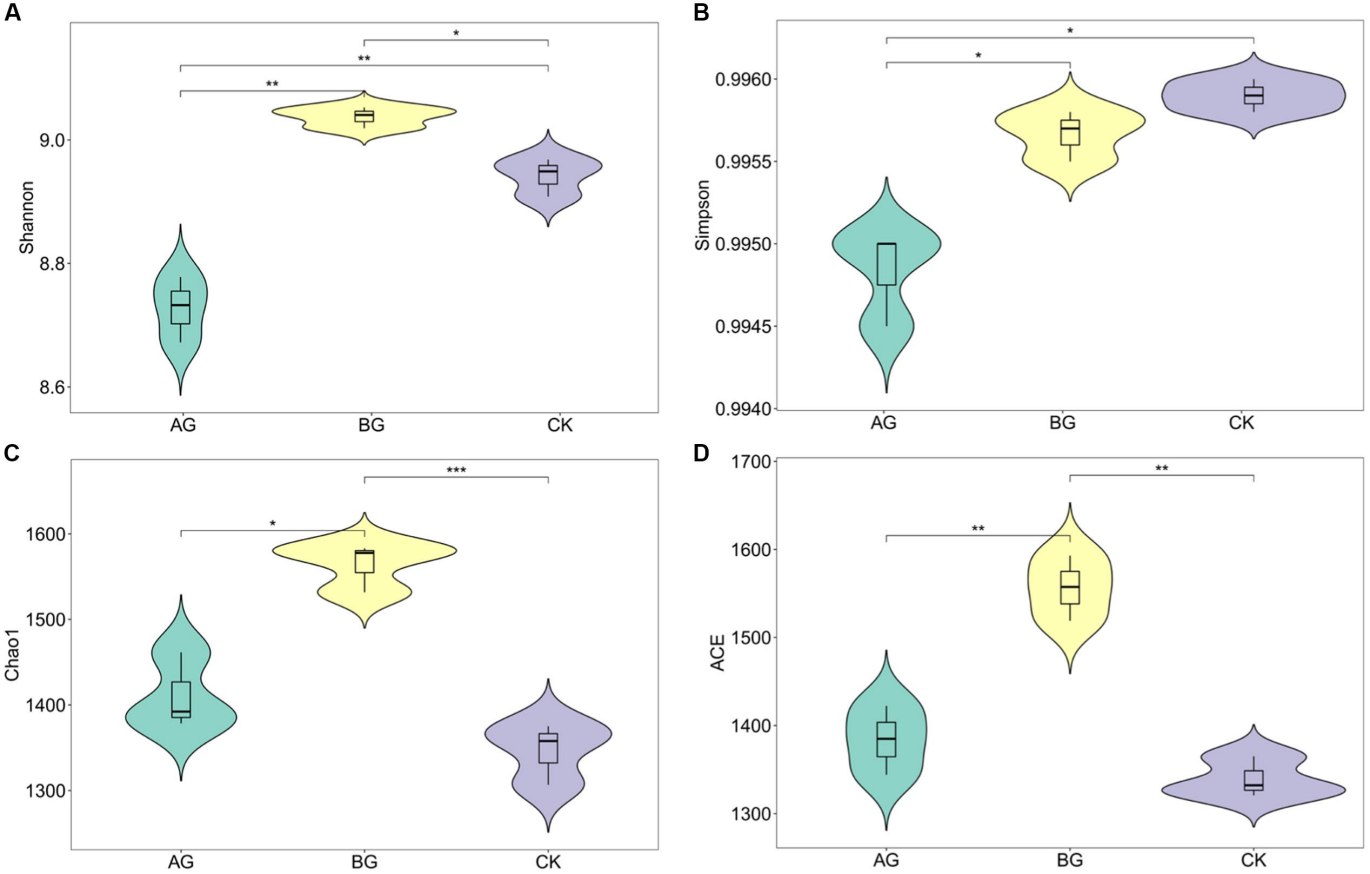
Shannon **(a)**, Simpson **(b)**, Chao1 **(c)** and ACE **(d)** in different treatments. The *, **, and *** respectively indicate a significant difference between means at *p* < 0.05, *p* < 0.01, and *p* < 0.001. CK denotes the treatment without gravel mulch (control), while AG and BG denote treatments with 2–5 mm and 40–60 mm particles used as gravel mulch, respectively.

The 6 years of gravel mulching significantly changed the soil microbial diversity indexes (Figure 3). The ACE and Chao1 indices showed a similar trend, being higher under the gravel mulching treatments than the control, and also significantly higher under the BG than AG treatment. Both Shannon and Simpson indexes of the AG treatment were significantly lower than the other treatments; the Shannon index of BG treatment was significantly higher than the control, but vice versa for the Simpson index. The ACE and Chao1 indexes are often used to estimate the total number of species (community richness); hence, we may infer that gravel mulching promotes the total number of microbial species in soil. The Shannon and Simpson indices are often used to represent microbial alpha diversity. These results indicate that the richness of soil microbial communities in the mulched treatment was improved compared to the control, although the increase of the AG treatment was not significant. Besides, the diversity of AG treatment was significantly lower than the other treatments. More soil microbial species after 6 years of gravel mulching could be explained by its ability to considerably increase soil temperature (Zhao et al., 2020). The discrepancy between the Shannon and Simpson diversity indexes of the large versus small particle-size treatments may be explained by their air permeability. The treatment using large gravel particles (BG) has a large pore size and a weak resistance to air evaporation resistance, whereas gravel mulching using small particles (AG) has a poor air permeability but a strong air resistance (Qiu et al., 2014), which further inhibits the decline of microbial species.

### 3.4. Analysis of soil bacterial and fungal community structure

**Figure 4:**
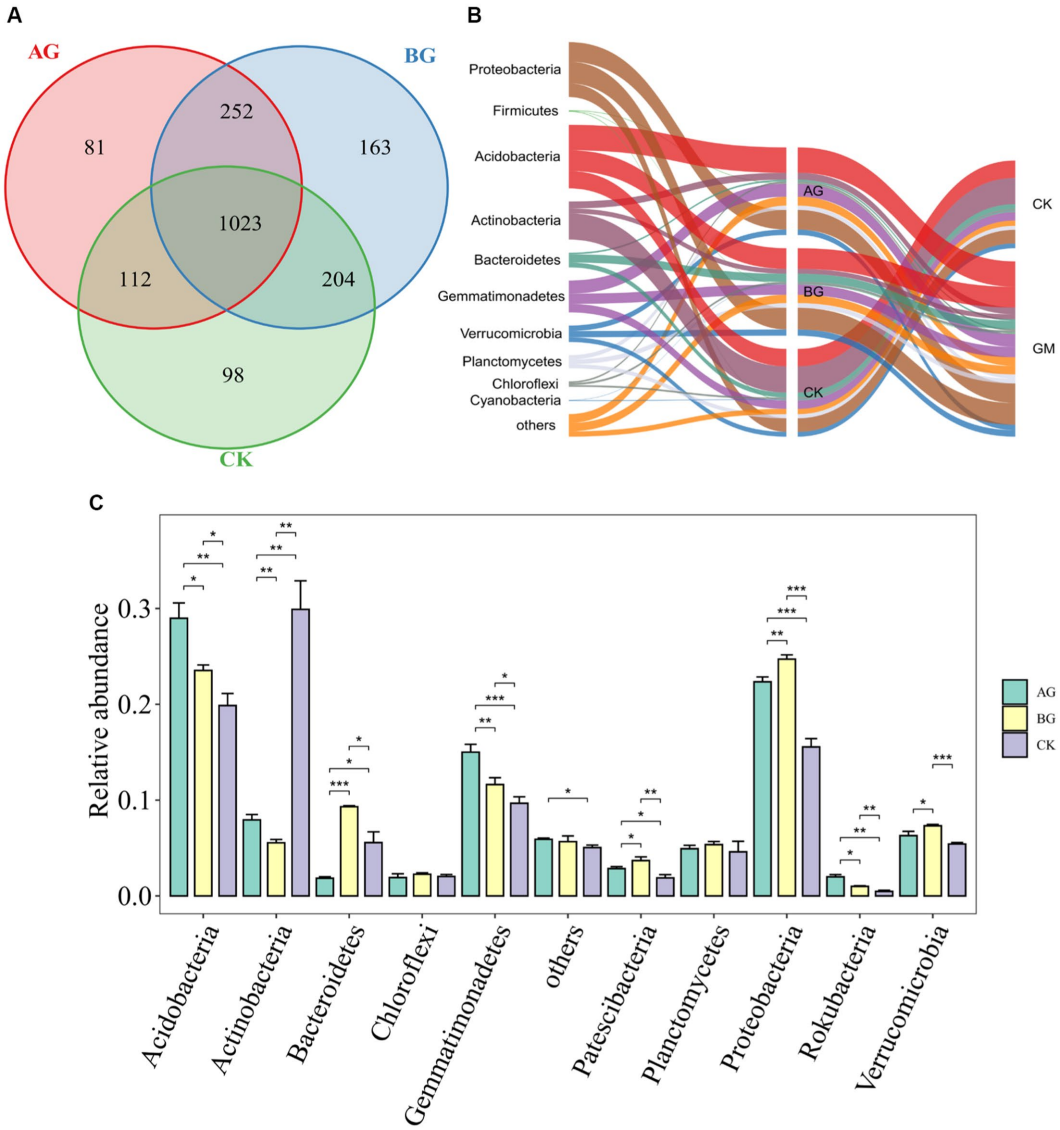
Venn diagram **(A)** Sankey diagram **(B)** and relative abundances of the main bacterial phyla **(C)** in soils from the three treatments. The *, **, and ***, respectively, indicate a significant difference between means at *p* < 0.05, *p* < 0.01, and *p* < 0.001. CK denotes the treatment without gravel mulch (control), while AG and BG denote treatments with 2–5 mm and 40–60 mm particles used as gravel mulch, respectively. GM represents the average level of gravel mulched treatment with different particle sizes.

A Venn plot (Figure 4A) showed that AG, BG and CK shared 1,023 bacterial community. There were 81, 163, and 98 unique bacterial community for AG, BG and CK, respectively. That also meant that AG and BG were more similar with each other, while CK showed less similarity with AG and BG. AG also had fewer unique compounds as compared with CK and BG. A sankey diagram (Figure 4B) showed the trend of relative abundance changes at the bacterial phylum level in different treatments, and Figure 4C shows the 6 years of gravel mulching resulted in a significant impact upon soil bacteria at the phylum level. According to the relative abundance distribution of detected phyla, *Acidobacteria* was the most abundant phylum in all samples (relative abundance of 18.5–30.8%); the other major dominant bacterial groups (i.e., relative abundance >5%) were *Proteobacteria*, *Actinobacteria*, *Gemmatimonadetes*, and *Verrucomicrobia*, having relative abundances of 14.9–25.1%, 5.2–31.6%, 18.5–30.8%, 9.0–15.9%, and 5.3–7.5%, respectively. As evinced by Figure 4, gravel mulching significantly changed the relative abundance of bacteria at the phylum level, by increasing that of *Acidobacteria* vis-à-vis the control, especially under the AG treatment, for which it was significantly higher than the other treatments. However, the relative abundance of *Actinobacteria* in either gravel mulching treatment was significantly lower than the control, while that in the BG treatment was significantly lower than the other two treatments. At the same time, the relative abundance of *Bacteroidetes* in the AG treatment was significantly lower than the other treatments, while that in BG treatment was significantly higher than the other treatments. For *Gemmatimonadetes*, its relative abundance was significantly higher under both gravel mulching treatments than the control, being significantly greater in the AG treatment than the other treatments. The relative abundance of *Proteobacteria* was also significantly higher under both gravel mulching treatments than the control, with that under the BG treatment being significantly greater than the other two treatments.

**Figure 5:**
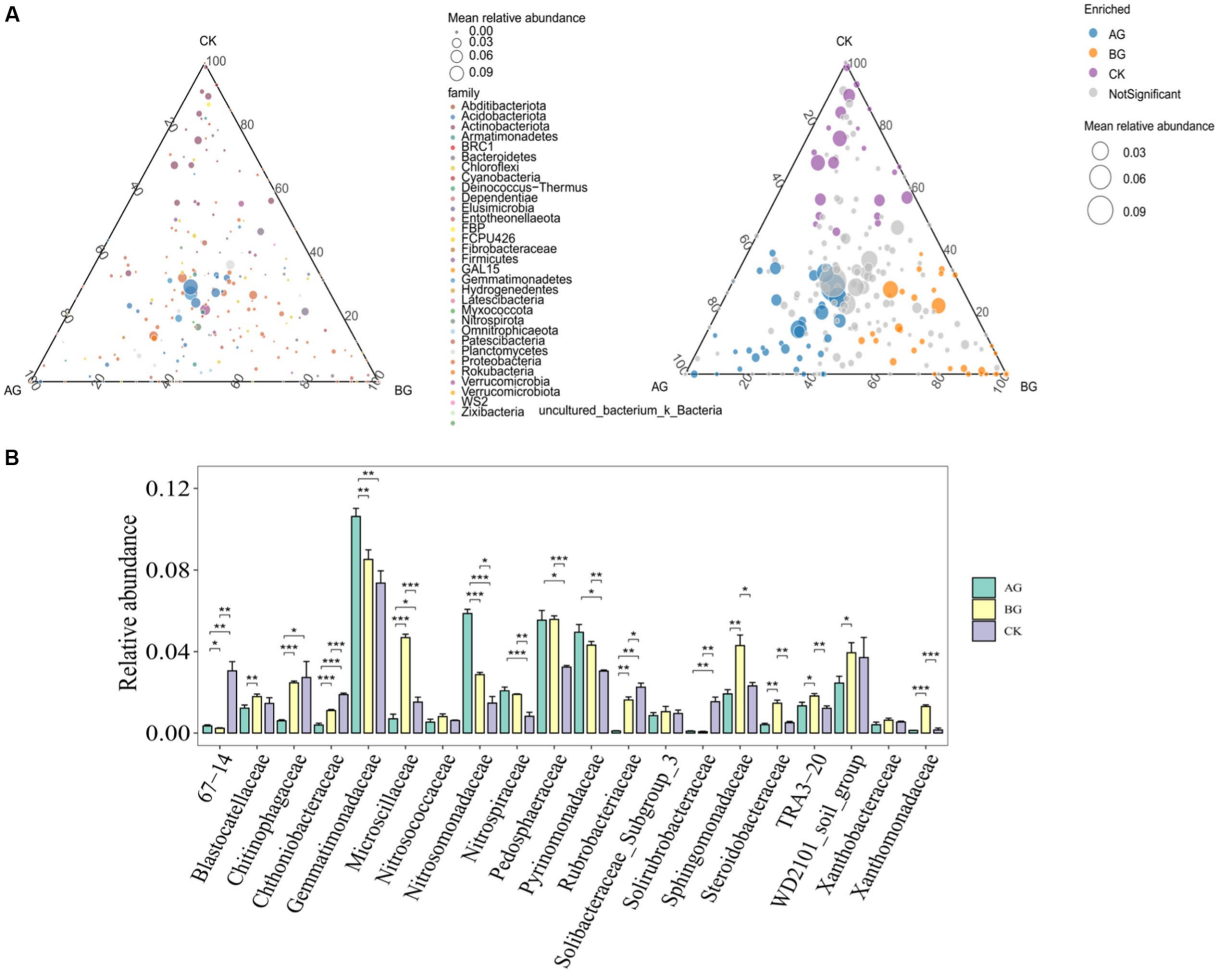
Ternary phase diagram **(A)** and relative abundances of the main bacterial family **(B)** in soils from the three treatments. The *, **, and ***, respectively, indicate a significant difference between means at *p* < 0.05, *p* < 0.01, and *p* < 0.001. CK denotes the treatment without gravel mulch (control), while AG and BG denote treatments with 2–5 mm and 40–60 mm particles used as gravel mulch, respectively.

From Figure 5, we can see the change in the relative abundances of various bacterial families under the different treatments. For example, while it was significantly increased by gravel mulching generally, the relative abundance of *Gemmatimonadaceae* in AG treatment was significantly higher than that in other two treatments; a similar trend was found for *Nitrosomonadaceae*. However, gravel mulching significantly decreased the relative abundance of certain bacterial families such as *Chitinophagaceae*, whose relative abundance under the AG treatment was significantly lower than that of other treatments.

**Figure 6:**
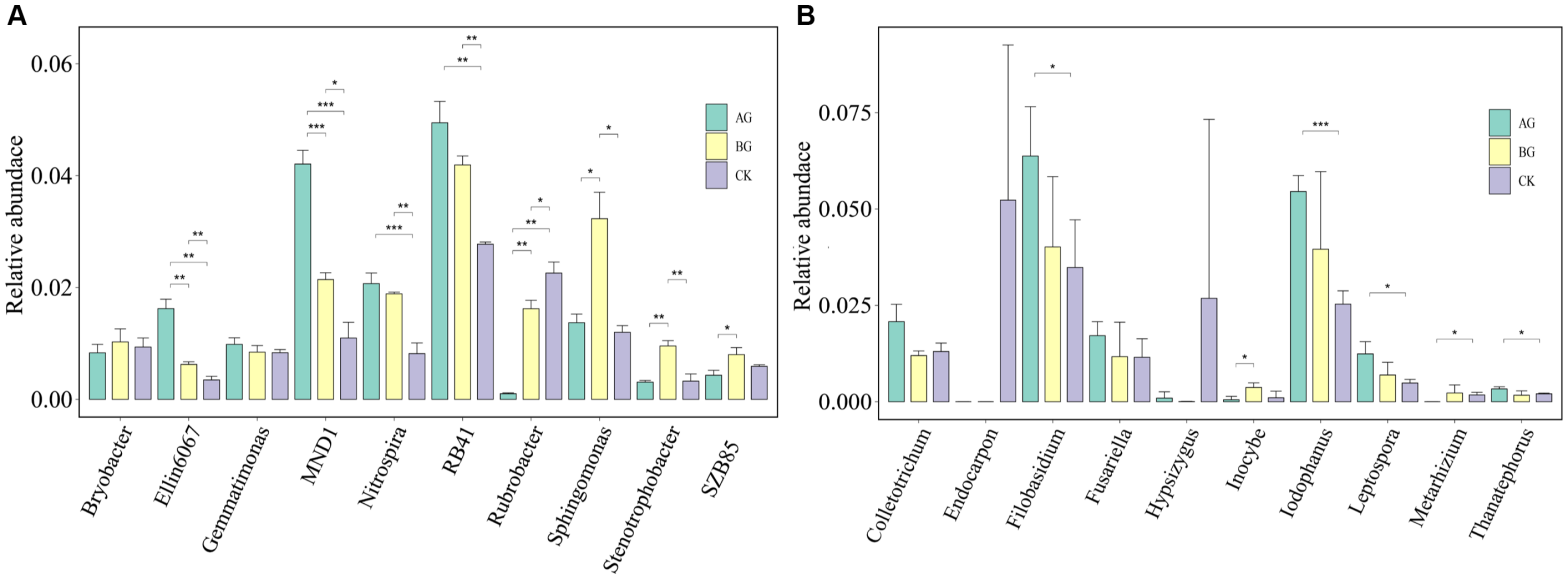
Relative abundances of the main bacterial genera **(A)** and fungal genera **(B)** in soils from the three treatments. CK denotes the treatment without gravel mulch (control), while AG and BG denote treatments with 2–5 mm and 40–60 mm particles used as gravel mulch, respectively. The *, **, and *** respectively indicate a significant difference between means at *p* < 0.05, *p* < 0.01, and *p* < 0.001.

Few species of fungi were detected in the plot soil, and the impact of gravel mulching on them at the phylum level was not significant, whereas significant gravel mulching-induced changes in the relative abundance of fungi at the genus level were found (Figure 6). For example, gravel mulching significantly increased the relative abundance of *Alternaria*, it being significantly higher in AG treatment than the other treatments. The relative abundances of *Fibulobasidium*, *Iodophanus* and *Torula* also showed similar trends. However, the relative abundance of *Metarhizium* featured a different trend, it being significantly reduced by the AG treatment compared with the control.

The bacterial and fungal community structure under the AG treatment differed from that under the BG treatment, which suggests gravel particle size is also related to the changed microbial community structure. After 6 years of gravel mulching, the soil nutrients of the AG and BG treatments had improved, and *Bacteroidetes* usually exist in environments having higher nutrient levels (Noah et al., 2007). An earlier study pointed out that *Proteobacteria* are usually abundant in soils with a high organic carbon content (Axelrood et al., 2002). In addition, the ratio of *Proteobacteria* and *Acidobacteria* can serve as an indicator to measure the soil fertility (Smit et al., 2001). In our study, compared with no mulching applied, *Proteobacteria* was more abundant in both gravel mulching treatments, whereas *Acidobacteria* was less abundant. This also shows that the soil nutrient level is greater after 6 years of gravel mulching than without it. In addition, the increased abundance of *Proteobacteria* and *Bacteroidetes* may be due to the enhanced elemental cycling, which in turn should augment soil fertility and thus promote plant growth (Lesaulnier et al., 2008). This could explain, in part, why gravel mulched farmland had significantly increased crop yields during the same cover time. *Alternaria* is a pathogenic fungus that can infect the leaves of melon crops. Previous researchers have reported on the leaf spot disease caused by *Alternaria* and the mechanism by which it renders melon seedlings white (Jia and Li, 2002; Lv et al., 2020). The main crops of gravel mulched farmland are melon crops, these dominated by watermelon. Therefore, that gravel mulching promotes the relative abundance of *Alternaria* is likely a chief reason for the characteristic decline in farmland production and land degradation that arises in the later period of gravel mulching.

**Figure 7:**
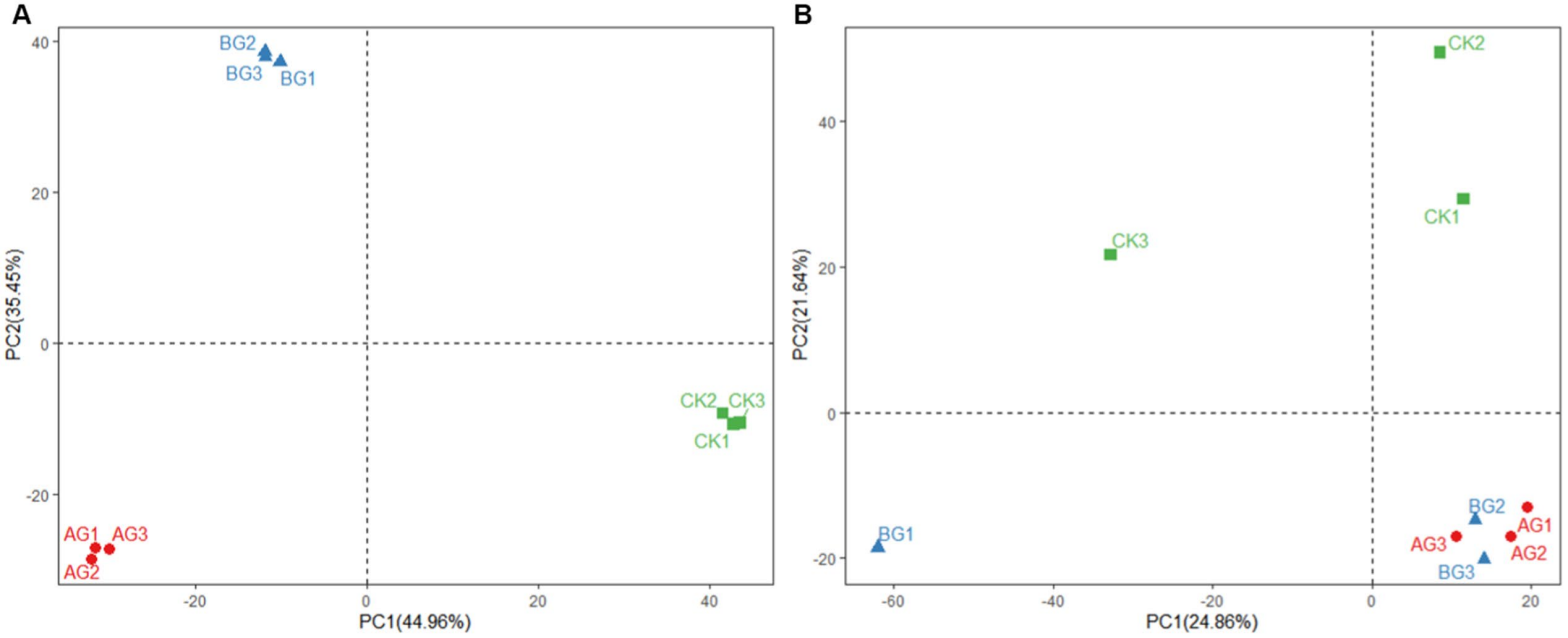
Principal component analysis (PCA) of bacterial **(A)** and fungal **(B)** communities in the three different treatments. CK denotes the treatment without gravel mulch (control), while AG and BG denote treatments with 2–5 mm and 40–60 mm particles used as gravel mulch, respectively.

**Figure 8:**
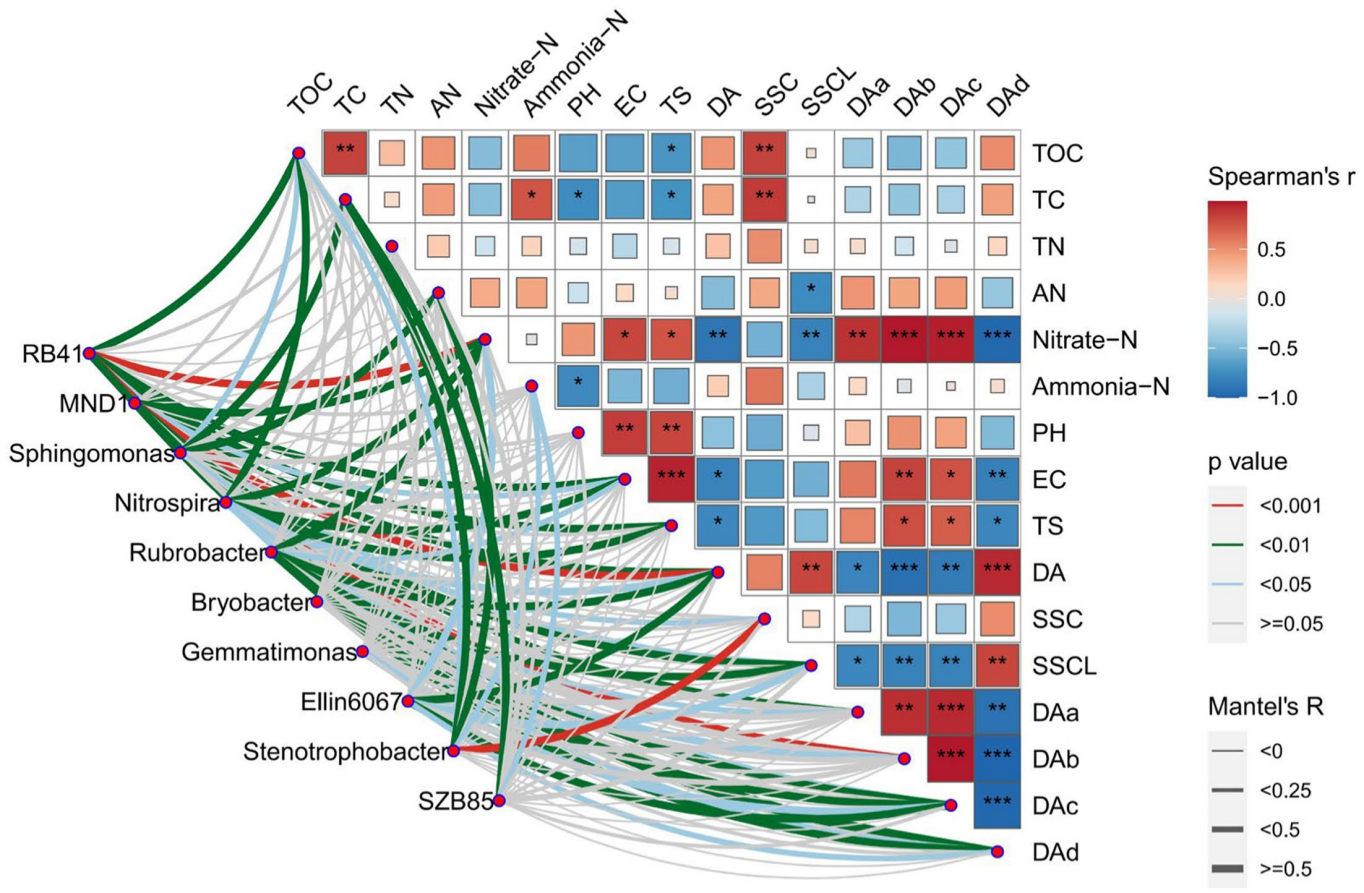
Mantel test of the main bacterial genera in soils from the three treatments. TC, total carbon; TOC, total organic carbon; TN, total nitrogen; AN, available nitrogen; TS, total salt; SSC, soil sand content; SSC, soil sand content >0.5 mm; DA, dust accumulation; DAa, dust accumulation with the particle size distribution of 0–7.8 μm; DAb, dust accumulation with the particle size distribution of 7.8–15.6 μm; DAc, dust accumulation with the particle size distribution of 15.6–31.2 μm; DAd, dust accumulation with the particle size distribution >31.2 μm. The *, **, and ***, respectively, indicate a significant difference between means at *p* < 0.05, *p* < 0.01, and *p* < 0.001. CK denotes the treatment without gravel mulch (control), while AG and BG denote treatments with 2–5 mm and 40–60 mm particles used as gravel mulch, respectively.

**Figure 9:**
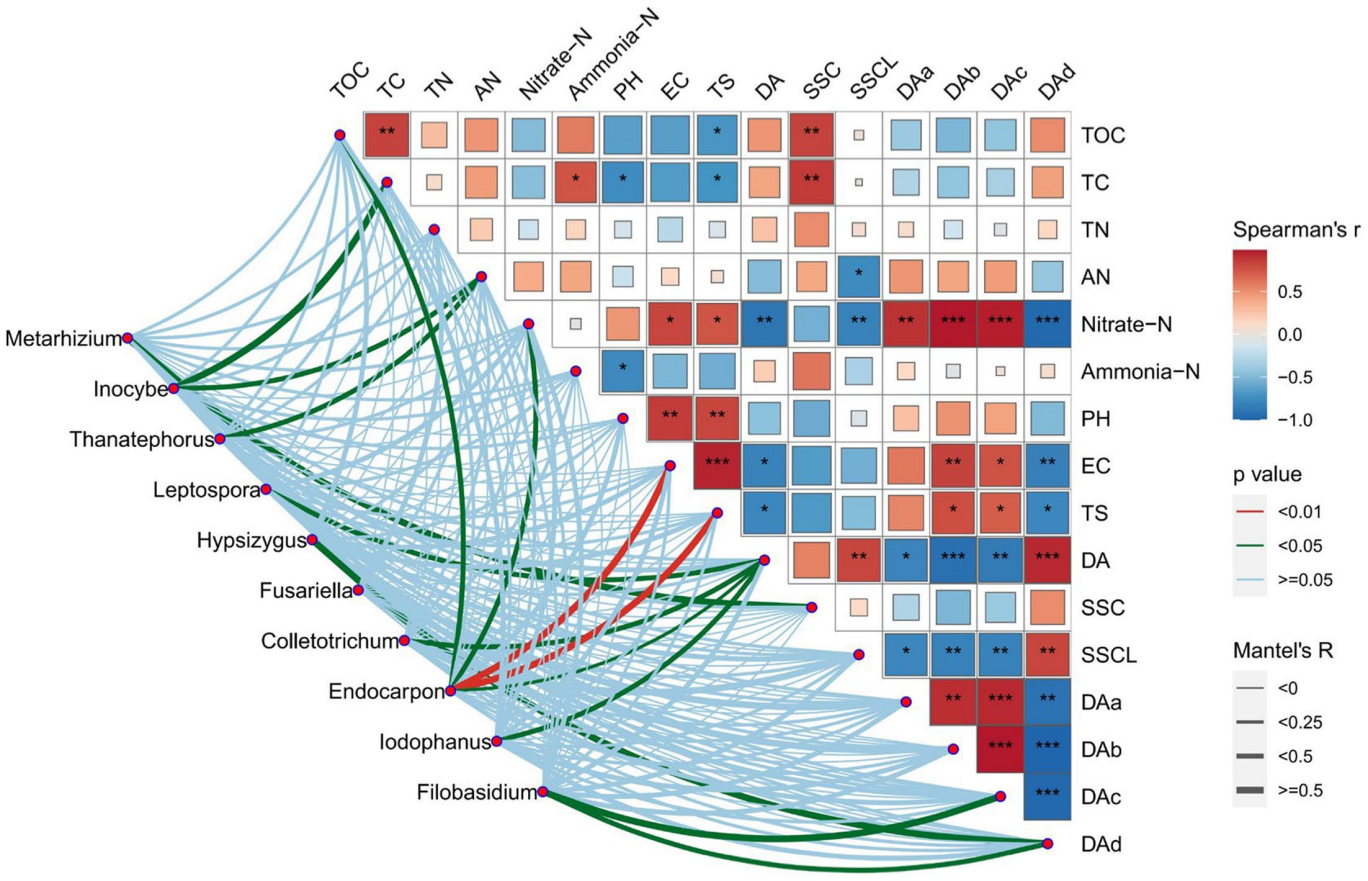
Mantel test of the main fungal genera in soils from the three treatments. TC, total carbon; TOC, total organic carbon; TN, total nitrogen; AN, available nitrogen; TS, total salt; SSC, soil sand content; SSC, soil sand content >0.5 mm; DA, dust accumulation; DAa, dust accumulation with the particle size distribution of 0–7.8 μm; DAb, dust accumulation with the particle size distribution of 7.8–15.6 μm; DAc, dust accumulation with the particle size distribution of 15.6–31.2 μm; DAd, dust accumulation with the particle size distribution >31.2 μm. The *, **, and ***, respectively, indicate a significant difference between means at *p* < 0.05, *p* < 0.01, and *p* < 0.001. CK denotes the treatment without gravel mulch (control), while AG and BG denote treatments with 2–5 mm and 40–60 mm particles used as gravel mulch, respectively.

PCA results for bacteria and fungal communities are shown in Figure 7. PCA was dissimilarity able to distinguish between the AG and CK bacteria communities from that of the BG treatment using the first principal component (PC1). The fungal community of the CK treatment differed in the first principal component (PC2) from those of the AG and BG treatments. PCA results showed that soil bacterial community structure under mulch treatments differed from those found in bare soil. Figures 8, 9 shows the effects of soil physical and chemical properties, DA in the mulching layer, and the sand content of surface soil on the top-10 genera of soil bacteria and fungi and the correlations among them. The sand content is significantly related to the content of TC and TOC in soil, while nitrate-N, TS, and EC in the soil are significantly related to DA in the gravel mulch layer. This indicates the importance of accumulating atmospheric dust as an external material source of soil under gravel mulching. The TC, TOC, AN, nitrate-N, and TS are soil physical and chemical properties greatly influenced the soil microbial community. For example, *Halophilic* microorganisms such as *Nitrilitruptoraceae* is very sensitive to changes in the concentrations of soil salt ions (Sorokin et al., 2009), and the abundance of *Endocarpon* is significantly correlated with salt ions in our study. This result is consistent with many previous studies finding that microbial community structure is affected by the nutrients in soil, such as carbon and nitrogen (Chodak et al., 2013; Delgado-Baquerizo et al., 2018). Given that soil nutrients are the material basis for the survival of soil microorganisms, changes to soil nutrient levels will affect the population growth of microorganisms, which may lead to changes in bacterial ecological strategies and ultimately changes in bacterial community composition over time and space (Noah et al., 2007; Huang et al., 2019). Organic carbon mainly modulates the structure of bacterial communities by regulating the energy and nutrients used by microorganisms in the process of nutrient transformation (Zhang et al., 2016). Among the various factors to affect soil microorganisms under gravel mulching, the capture of dust by gravel mulch and the modified carbon and nitrogen components in the soil evidently play a major driving role. Soil sand content may have altered specific heat, changing soil temperature and evaporation, thereby affecting soil microorganisms. Yet, considering that improving soil sand content in the soil is a process of gradual mixing of the overburden and the soil surface, and that changing both soil salt and pH is also a long-term process, more long-term observations and experimental research are needed to distinguish and gage their roles on longer temporal scales. In this context then, the results of this study can enhance our understanding of the relations between microbial community composition and soil property changes. Nonetheless, we still need more long-term studies from other sites to reveal the underlying mechanisms that drive pronounced and subtle changes in microbial community composition under gravel mulching.

## 4. Conclusion

Gravel mulched farmland has been widely used in the semi-arid region of northwest China because it can increase soil temperature and retain water and reduce evaporation. But in recent years, its long-term coverage has led to soil degradation whose causes are still unclear. From the field experiment’s results, we may draw the following conclusions: (1) After 6 years of gravel mulching, the topsoil (0 to 20 cm) under gravel mulching contained significantly higher TC and TOC concentrations than non-mulched soil. (2) Six years of gravel mulching significantly changed the soil microbial diversity and re-shaped the abundance distribution of bacterial and fungal communities. These effects were caused by differing physical and chemical properties of soil and the differential capture capacity of atmospheric dust by gravel mulch layers with small versus large particles. (3) Among various factors examined, on the 6-year scale, the capture of dust by gravel mulching and the change to soil carbon and nitrogen both play a major driving role in affecting soil microorganisms.Our results suggest the change via gravel mulching to soil microorganisms may be the key factor leading to subsequent soil degradation and the effects of different particle size covers on dust reduction, soil physicochemical properties, and soil microorganisms are not the same. Therefore, a more reasonable mulching layer structure may slow down the process of soil degradation. Finally, more long-term, well-replicated studies are needed to reveal the underlying mechanisms that drive soil degradation under gravel mulching.

## Data Availability Statement

The datasets presented in this study can be found in online repositories. The names of the repository/repositories and accession number(s) can be found below: https://www.ncbi.nlm.nih.gov/genbank/, Mendeley Data, V1, doi: 10.17632/mfsyk6y94m.1.

## Author Contributions

YQ: formulation of overarching research goals and aims. XC: methodology. YW: performed the data analysis. YZ: validation. ZX: project administration. All authors contributed to the article and approved the submitted version.

## Conflict of interest

The authors declare that the research was conducted in the absence of any commercial or financial relationships that could be construed as a potential conflict of interest.

## Publisher’s note

All claims expressed in this article are solely those of the authors and do not necessarily represent those of their affiliated organizations, or those of the publisher, the editors and the reviewers. Any product that may be evaluated in this article, or claim that may be made by its manufacturer, is not guaranteed or endorsed by the publisher.
